# Polyploid Giant Cancer Cells as a Senescence-Linked State in the Tumor Microenvironment

**DOI:** 10.3390/cancers18111683

**Published:** 2026-05-22

**Authors:** Michelle R. Dawson, Deepraj Ghosh

**Affiliations:** 1 Department of Molecular Biology, Cell Biology, and Biochemistry, Brown University, Providence, RI 02912, USA; 2 Institute for Biology, Engineering and Medicine, Brown University, Providence, RI 02912, USA

**Keywords:** cellular senescence, senescence-associated secretory phenotype (SASP), tumor microenvironment, extracellular matrix remodeling, polyploid giant cancer cells (PGCCs), tumor dormancy, therapy resistance, mechanobiology, tumor recurrence

## Abstract

Cellular senescence and polyploidy are fundamental stress responses that shape cancer progression and therapeutic outcomes. While senescence initially suppresses tumor growth, senescent cells accumulate in aging and therapy-exposed tissues and actively remodel the tumor microenvironment through the senescence-associated secretory phenotype (SASP) and extracellular matrix (ECM) reorganization. Senescent stromal cells increase collagen deposition and generate disordered matrix architectures, as evidenced by enhanced second harmonic generation (SHG) signal and increased anisotropic variation across in vitro systems, 3D co-culture models, and fibrotic lung tissues. These biochemical and mechanical alterations promote cancer cell plasticity and create conditions permissive for disease progression. Polyploid giant cancer cells (PGCCs) are a rare but highly resilient cancer cell population enriched under genotoxic stress. PGCCs arise through mitotic failure, including mitotic slippage and cytokinesis defects, and can survive chemotherapy and radiation due to their altered cell-cycle regulation. Emerging evidence indicates that senescence-driven microenvironments promote the formation of PGCCs and multinucleated cells, linking ECM remodeling and mechanical stress to polyploidization. Functionally, PGCCs exhibit abnormal cytoskeletal and nuclear mechanics that support migratory persistence and enable survival within hostile tumor environments. In addition, PGCCs can promote the survival of neighboring cancer cells during treatment, suggesting a stromal-like role in establishing therapy-resistant niches. These cells can persist in a dormant state and later generate proliferative progeny, contributing to tumor recurrence and metastasis. Together, these findings support a model in which senescent niches may promote PGCC formation, persistence, and tumor repopulation. Targeting both senescence-associated microenvironments and PGCC-specific survival mechanisms may improve long-term therapeutic outcomes.

## Simple Summary

Cellular senescence and polyploidy are adaptive stress responses that influence cancer progression, therapy resistance, and disease recurrence. While senescence initially suppresses tumor growth by halting proliferation, senescent stromal cells accumulate in aged and therapy-exposed tissues and develop a secretory phenotype that remodels the tumor microenvironment. These cells also deposit disorganized and mechanically stiff extracellular matrix, generating biochemical and mechanical signals that reshape cancer-cell behavior. Emerging evidence suggests that these senescence-associated niches promote the formation of polyploid giant cancer cells, rare cell populations characterized by extreme size, genomic instability, dormancy, and the ability to generate aggressive progeny through amitotic budding. This review synthesizes current evidence linking senescence-associated microenvironment remodeling to the emergence of polyploid cells and highlights how the mechanics of these cells contribute to therapy resistance and metastatic dissemination. Understanding polyploid giant cancer cells as a senescence-linked state may reveal new therapeutic strategies to eliminate dormant cancer cells and prevent relapse.

## 1. Introduction

Cellular senescence and polyploidy represent two fundamental responses to cellular stress that influence cancer progression and therapeutic outcomes [1,2]. Senescence has long been viewed as a tumor-suppressive mechanism that halts the proliferation of damaged cells. However, senescent cells accumulate in aging tissues [3] and in tumors exposed to chemotherapy or radiation [4,5], where they actively remodel the surrounding microenvironment through the senescence-associated secretory phenotype (SASP) [6]. These changes alter inflammatory signaling, extracellular matrix organization, and the mechanical properties of the tumor niche.

At the same time, rare populations of polyploid giant cancer cells (PGCCs) are increasingly recognized in aggressive and therapy-resistant tumors [7,8]. PGCCs are characterized by extreme cellular enlargement, genomic instability, and the ability to survive cytotoxic stress while remaining metabolically active. Although PGCCs may persist in a dormant-like state similar to senescent cells, they retain the capacity to re-enter the cell cycle and generate proliferative progeny that can repopulate tumors after treatment [9]. Increasing attention has focused on the idea that polyploidy may represent an adaptive stress-response program that shares biological features with therapy-induced senescence (TIS) and dormancy-associated states, while still retaining the potential for regenerative escape and tumor recurrence [10]. In parallel, mechanobiological remodeling of the tumor microenvironment is emerging as an important regulator of both senescence and PGCC biology, including extracellular matrix stiffening, altered cytoskeletal signaling, and stress-associated inflammatory programs [11,12].

Growing evidence suggests that these phenomena may be mechanistically linked [13]. Senescence-driven remodeling of the tumor microenvironment may create biochemical and mechanical conditions that promote the emergence and persistence of PGCCs [12]. Understanding how senescent niches contribute to PGCC formation may therefore provide new insight into the mechanisms underlying therapy resistance, tumor dormancy, and disease recurrence. In this review, we examine how senescence reshapes the tumor microenvironment, discuss the biological and mechanobiological features of PGCCs, and highlight emerging evidence linking senescence-associated niche remodeling to polyploid stress adaptation, therapy resistance, tumor dormancy, and recurrence.

## 2. Senescence-Driven Remodeling of the Tumor Microenvironment

Chronological age is the leading risk factor for developing cancer, and many anticancer therapies, including radiation and chemotherapy, can also induce senescence in stromal and epithelial cells [14]. Even low-dose radiation triggers sustained cell-cycle arrest and prolonged expression of cell-cycle inhibitors such as p16 and p21 in normal stromal cells, including stem cell populations [15]. Although senescence initially suppresses tumor growth by halting proliferation, senescent cells can persist in tissues and acquire a senescence-associated secretory phenotype (SASP) that is marked by sustained secretion of pro-inflammatory cytokines, growth factors, extracellular matrix (ECM) components, and matrix-remodeling enzymes [6]. Collectively, these features promote tissue environments characterized by chronic inflammation and fibrosis [16,17].

SASP signaling profoundly alters the tumor microenvironment. Senescent stromal cells recruit and reprogram surrounding cells, enhance cancer cell motility and invasiveness, and reshape the overall tissue architecture by inducing matrix stiffening and reorganization [18]. Senescent fibroblasts have been shown to accelerate tumor growth in vivo [19,20], promote tumor dormancy and therapy resistance [5], and induce epithelial-to-mesenchymal transition (EMT) in otherwise non-motile breast cancer cells [21,22].

Beyond secretory signaling, senescent cells remodel the extracellular matrix (ECM) in ways that directly influence cancer cell behavior. In our prior studies, senescence was induced in bone marrow–derived marrow stromal cells (HMSCs) and lung fibroblasts (LF1) using 15 Gy γ-irradiation. Senescence was confirmed by increased β-galactosidase activity, upregulation of p16, p21, and APO1, and reduced proliferation (Table 1) [18,23].

**Figure 1:**
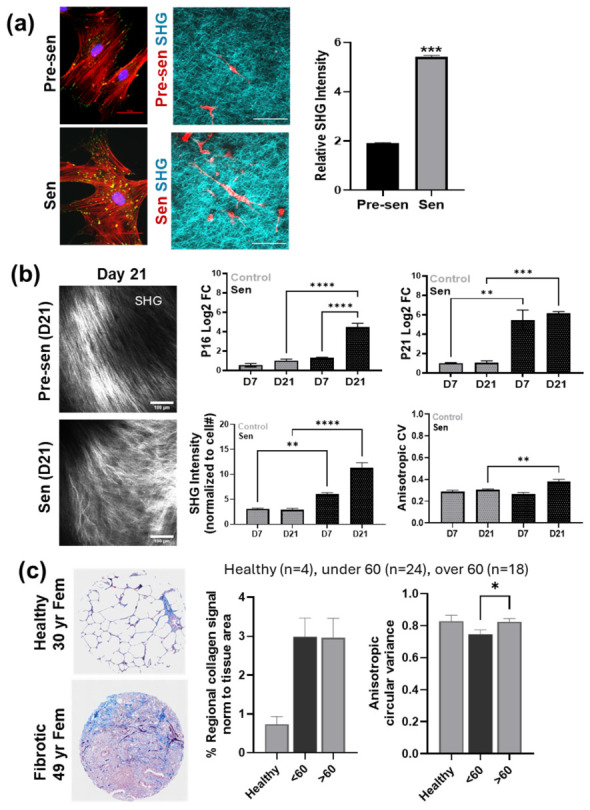
Senescence-driven extracellular matrix remodeling in vitro and in vivo. (**a**) Pre-senescent and senescent MSCs stained for F-actin (red), vinculin (green), and nuclei (blue) exhibit disrupted cytoskeletal organization and more randomly distributed focal adhesions in senescence. Multiphoton microscopy of MSCs (red) embedded in 3D collagen gels (SHG, blue) shows that senescent MSCs deposit increased amounts of disorganized collagen. (**b**) Senescent LF1 fibroblasts cultured in fibroblast-derived matrices display increased p16 and p21 expression by day 21. SHG imaging reveals increased normalized collagen signal and higher anisotropic circular variance, indicating greater collagen deposition and reduced fiber alignment in senescence. (**c**) Lung tissue arrays, including 4 healthy controls and 42 pulmonary fibrosis biopsies, were analyzed for collagen using Masson’s trichrome staining. Fibrotic samples exhibited increased collagen deposition, with tissues from donors over 60 showing higher anisotropic circular variance, indicative of more disordered collagen architecture. All assays were performed with a minimum of three replicates and results are presented as mean ± SEM. Statistical significance was determined using Student’s *t*-test for two-group comparisons or ANOVA for multiple-group comparisons (* *p*  <  0.05, ** *p*  <  0.01, *** *p*  < 0.001, **** *p*  <  0.001). Parts of this figure were reproduced and modified from Ghosh et al., Journal of Cell Science (2020) [18], with permission from The Company of Biologists and Howes et al., APL Bioengineering (2024) [23], which was distributed under a Creative Commons Attribution (CC BY) license.

Senescent cells also exhibited increased cell and nuclear areas and reduced motility, consistent with a senescent phenotype (summarized in Table 1). Importantly, these cells also deposited and crosslinked ECM proteins, altering the architecture and mechanical properties of collagen-rich environments (Figure 1a); this ECM remodeling phenotype was associated with more disordered pattern of actin filaments and focal adhesion proteins in the cytoskeleton [18,23].

Using fibroblast-derived matrix models, we further observed that senescence increases the deposition of highly disordered collagen fibers (Figure 1b). This was associated with alterations in integrin expression and more heterogeneous cytoskeletal organization with multilaterally distributed cytoskeletal tension. Similar patterns of increased senescence (based on elevated p16 staining) and disordered collagen deposition (reflected by increased anisotropic circular variance) were observed in aging human lung tissues from pulmonary fibrosis patients (Figure 1c), indicating that senescence-associated ECM remodeling occurs in vivo and is not merely an artifact of in vitro culture [23].

**Figure 2:**
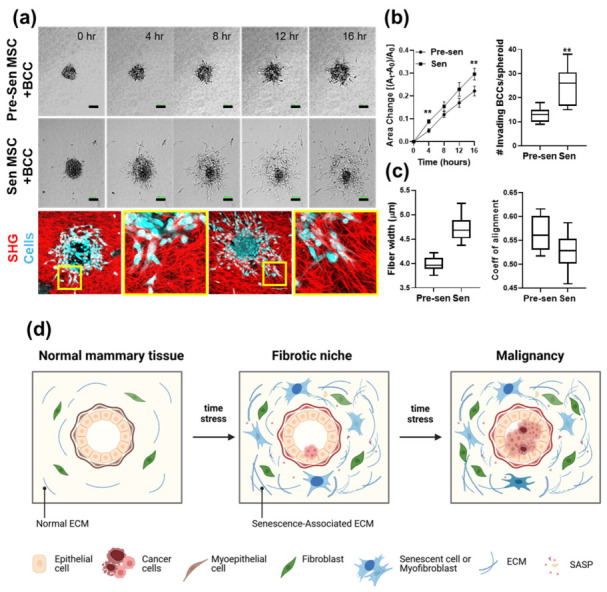
Senescent cells promote breast cancer invasion through ECM remodeling. Spheroids composed of equal numbers of marrow stromal cells (MSCs) and MDA-MB-231 breast cancer cells (BCCs) were embedded in 3D collagen gels. ((**a**), **top**) Time-lapse imaging over 16 h was used to quantify BCC invasion from spheroids containing pre-senescent or senescent MSCs. (**b**) Invasion was quantified as area normalized to initial spheroid size and as the number of invading BCCs per spheroid. ((**a**), **bottom**) Multiphoton microscopy with second harmonic generation (SHG) was used to visualize collagen fibers (red) and NucRed-labeled cells (blue). Zoomed regions of cells migrating into surrounding collagen are highlighted in yellow. (**c**) Co-culture with senescent MSCs increased collagen fiber width and decreased alignment, indicating thicker and more disorganized ECM. All assays were performed with a minimum of three replicates and results are presented as mean ± SEM. Statistical significance was determined using Student’s *t*-test for two-group comparisons or ANOVA for multiple-group comparisons (** *p*  <  0.01). This figure was reproduced and modified from Ghosh et al., Journal of Cell Science (2020) [18], with permission from The Company of Biologists. (**d**) Schematic illustrating how senescence-associated ECM remodeling within a fibrotic niche promotes tumor progression. Created in BioRender. Dawson, M. (2026) https://BioRender.com/4pr089v (27 March 2026).

While increased extracellular matrix (ECM) stiffness has been linked to breast cancer progression [24], the contribution of senescent cells has remained less well defined [25]. Using single-cell analyses and a 3D matrix interface model, we characterized the biophysical properties of senescent marrow stromal cells (MSCs) and their influence on cancer cell behavior [18]. Although senescent MSCs exhibited reduced motility compared to pre-senescent cells, they promoted a more invasive breast cancer phenotype, marked by enhanced spheroid growth and breast cancer cell invasion of the collagen matrices (Figure 2a,b). Second harmonic generation imaging further revealed increased collagen density and matrix disorder in co-cultures with senescent MSCs (Figure 2a,c), indicating active remodeling of the extracellular environment. Together, these findings provide direct evidence that senescent MSCs drive pro-malignant remodeling of the tumor microenvironment [18]. This process is illustrated in Figure 2d.

Integrin signaling plays a key role in linking these matrix changes to cellular behavior. Integrin αvβ3, which is upregulated in aging and senescent cells, mediates TGFβ activation and contributes to the senescent secretory phenotype [26,27]. We previously demonstrated that inhibition of the TGFβ signaling pathway reduces senescence-associated ECM remodeling [23]. Integrins also cluster to form focal adhesions that connect the actin cytoskeleton to the ECM and activate intracellular signaling pathways including FAK and Src, thereby integrating mechanical and biochemical cues that regulate cell survival, migration, and growth [28]. In parallel, senescent cells release increased numbers of extracellular vesicles (EVs) containing factors that can activate TGFβ signaling and drive fibroblast-to-myofibroblast differentiation, thereby amplifying ECM remodeling [29].

These combined biochemical and mechanical signals reshape the tumor microenvironment and create conditions that promote cancer cell plasticity [30]. Increased ECM disorder and stiffness enhance integrin engagement, elevate intracellular tension, and alter cytoskeletal organization in adjacent cancer cells [18].

Mechanical signals generated by senescence-associated ECM remodeling are transmitted intracellularly through integrin-mediated focal adhesion complexes involving focal adhesion kinase (FAK), Rho GTPases, and downstream actomyosin signaling pathways [31]. FAK functions as a central mechanosensory scaffold that responds to increased ECM stiffness by activating signaling cascades including RhoA–ROCK, PI3K/Akt, and ERK pathways, which collectively regulate cytoskeletal remodeling, intracellular tension, and stress survival [32]. Rho GTPases including RhoA, Rac1, and Cdc42 coordinate actin dynamics, spindle organization, and cellular contractility in response to mechanical stress.

These pathways are particularly relevant in the context of therapy-induced stress because dysregulated actomyosin contractility and spindle assembly can promote mitotic slippage, cytokinesis failure, tetraploidization, and survival of polyploid stress-adapted cells [33,34]. Among downstream mechanotransduction mediators, YAP/TAZ represents one of the best-characterized transcriptional effectors linking ECM stiffness and cytoskeletal tension to cellular plasticity and stress adaptation [35]. YAP/TAZ activation is regulated by focal adhesion signaling, RhoA-dependent actin remodeling, and intracellular mechanical tension, and has been associated with transcriptional programs linked to stemness, survival plasticity, and therapy resistance [36]. Together, these findings suggest that senescence-associated ECM remodeling may promote PGCC formation not only through inflammatory signaling, but also through mechanically induced alterations in cytoskeletal organization and mitotic fidelity.

Importantly, these mechanically altered niches increase the frequency of multinucleated and polyploid cells, suggesting that senescence-associated remodeling of the tumor microenvironment creates a permissive niche for cancer progression and the emergence of polyploid giant cancer cells [13].

## 3. Polyploid Giant Cancer Cells: Formation and Biological Features

Polyploidy is increasingly recognized as an adaptive stress-response program that emerges in response to aging, chronic inflammation, hypoxia, genotoxic injury, and cancer therapy [37]. In many contexts, low-ploidy or tetraploid states may initially function as protective mechanisms that preserve cell viability under conditions of severe stress by limiting proliferation and buffering against stress-induced cell death [38]. Consistent with this concept, polyploid cells accumulate in aging tissues across multiple organ systems, including liver, vascular, epithelial, and brain tissues, and experimentally induced tetraploid cells frequently exhibit prolonged growth arrest and senescence-associated characteristics [39,40,41]. These observations suggest that stable polyploid states may initially function as dormancy- or senescence-like adaptations that allow cells to survive otherwise lethal stress conditions.

**Figure 3:**
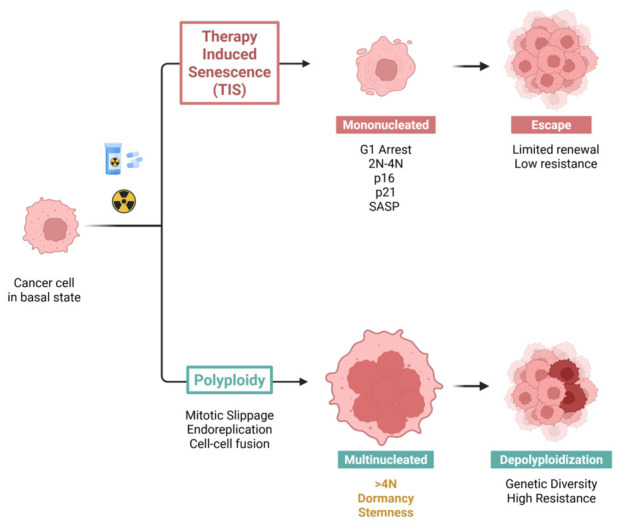
Stress-Induced Transitions Between Therapy-Induced Senescence, PGCC Formation, and Regenerative Escape. Genotoxic and microenvironmental stressors, including chemotherapy and radiation, can induce therapy-induced senescence (TIS) or promote polyploid giant cancer cell (PGCC) formation through mitotic slippage, endoreplication, or cell fusion. TIS and PGCCs share several stress-associated features, including persistent DNA damage signaling, p21 or p16 induction, SASP-associated cytokine secretion, and prolonged cell-cycle arrest or dormancy-like states. However, unlike canonically senescent cells, PGCCs retain the capacity for regenerative escape through depolyploidization and neosis. PGCC-derived daughter cells can subsequently re-enter the cell cycle, acquire therapy resistance, and contribute to tumor repopulation and genetic diversity. Created in BioRender. Ghosh, D. (2026) https://BioRender.com/lwq297d (9 May 2026).

However, chronic stress exposure and dysregulated cell-cycle control can promote progression from stable tetraploid arrest toward more plastic and regenerative polyploid states associated with PGCC formation [41]. Both therapy-induced senescence (TIS) and PGCCs emerge following genotoxic stress and share several stress-associated features, including persistent DNA damage signaling, p21 induction, SA-β-gal activity, SASP-associated cytokine secretion, and prolonged cell-cycle arrest [42]. Importantly, unlike canonically senescent cells, PGCCs retain the capacity to undergo depolyploidization and regenerative escape through neosis, an asymmetric cell division process in which PGCCs undergo nuclear budding followed by asymmetric cytokinesis to generate diploid or aneuploid progeny cells capable of proliferative re-entry following therapy-induced stress [9]. This transition from stress-associated arrest toward regenerative polyploid states may contribute to tumor dormancy escape, acquisition of therapy resistance, and disease recurrence. These transitions between therapy-induced senescence, PGCC formation, and neosis-associated regenerative escape are illustrated in Figure 3.

PGCCs have long been recognized histopathologically as large, atypical multinucleated cells enriched in high-grade tumors and in cancers exposed to chemotherapy or radiation [43,44]. They are particularly prominent in triple-negative breast cancers and metastatic lesions [43,45]. PGCCs contain excess chromosomes, exhibit chromosomal instability, and display highly abnormal nuclear and cytoskeletal organization [46]. Functionally these cells can persist in stress-adapted or dormant-like states while remaining metabolically active and later re-enter the cell cycle through amitotic budding, generating genetically diverse and therapy-resistant progeny [9].

Multiple mechanisms contribute to PGCC formation. Mitotic slippage allows cells to exit mitosis without completing division when spindle checkpoints fail, whereas endoreplication enables DNA synthesis without mitosis or cytokinesis [47]. Inflammatory microenvironments can also promote cell fusion events that generate multinucleated cells [48]. These processes are closely linked to dysregulation of cell-cycle regulators and mitotic kinases such as the Aurora kinase family [49]. Aurora A overexpression promotes centrosome amplification, chromosomal instability, and tetraploidization, while inhibition of Aurora B disrupts chromosome segregation and induces polyploidy [49]. Dysregulation of these pathways is common in aggressive cancers and contributes to chromosomal instability, polyploidization, and therapeutic resistance [50]. Accordingly, Aurora kinase inhibitors are being investigated as therapeutic strategies for targeting mitotic dysregulation and survival of PGCCs.

**Figure 4:**
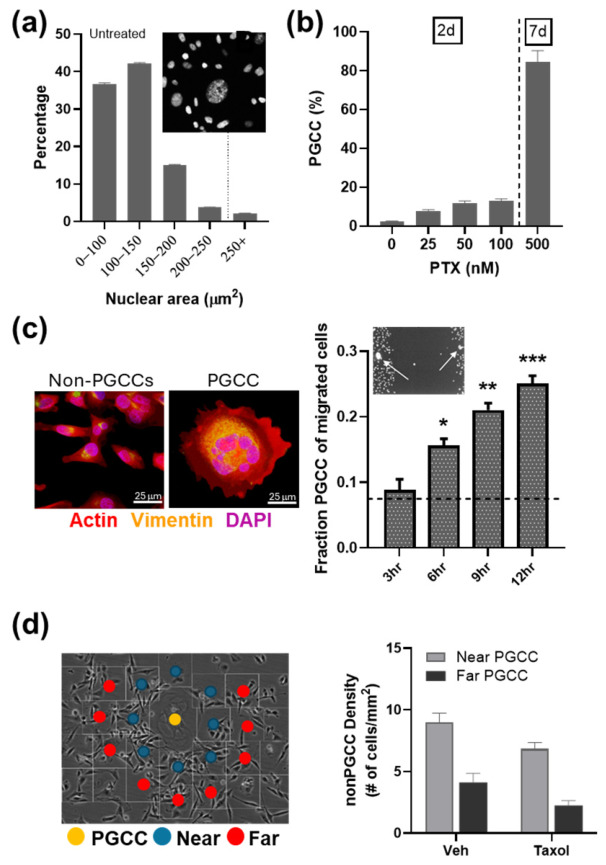
PGCC enrichment and stromal-like interactions promote survival and migration. (**a**,**b**) PGCCs were identified in Hoechst-stained images of untreated (**a**) and DMSO- or paclitaxel (PTX)-treated MDA-MB-231 cells (**b**) based on nuclear area ≥2.5× the population mean. The dashed line in (**a**) represents the cutoff used to define PGCCs. PTX treatment (500 nM, 7 days) enriched PGCCs to ~90% of the cell population. The dashed line in (**b**) separates data collected at different time points. (**c**) PGCCs stained for actin (red), vimentin (orange), and nuclei (fuchsia) exhibit reorganized cytoskeletal networks with more dispersed actin and vimentin, supporting increased migratory persistence. Scratch assays show preferential migration and enrichment of PGCCs within the wound region over time. The dashed line indicates the fraction of PGCCs present in the cell population prior to scratch formation. (**d**) Spatial analysis during PTX treatment shows enhanced survival of non-PGCCs in close proximity (blue) to PGCCs (orange) compared to more distant cells (red), indicating that PGCCs likely promote local survival of neighboring cancer cells. All assays were performed with a minimum of three replicates and results are presented as mean ± SEM. Statistical significance was determined using Student’s *t*-test for two-group comparisons or ANOVA for multiple-group comparisons (* *p*  <  0.05, ** *p*  <  0.01, *** *p*  <  0.001). Parts of this figure were reproduced and modified from Xuan et al., Scientific Reports (2018) [12] and Xuan et al., Proc. Natl. Acad. Sci. U.S.A. (2020) [51], both were distributed under Creative Commons Attribution (CC BY) licenses.

Therapeutic and microenvironmental stress strongly enrich PGCC populations by eliminating proliferative competitors and selecting for stress-adapted polyploid states [43,44]. Consistent with this, paclitaxel (PTX) treatment rapidly increased the proportion of PGCCs in multiple cell lines in our studies [12,51,52]. While PGCCs comprised ~2% of untreated MDA-MB-231 cells (Figure 4a), they increased to ~9% of surviving cells 24–48 h following 100 nM PTX and dominated the populations (~90%) within 7 days of 500 nM PTX treatment (Figure 4b). These surviving PGCCs subsequently generated proliferative daughter cells with increased resistance to PTX, highlighting their role as reservoirs of cancer cells that can repopulate tumors after therapy and drive chemoresistance [45,46].

**Figure 5:**
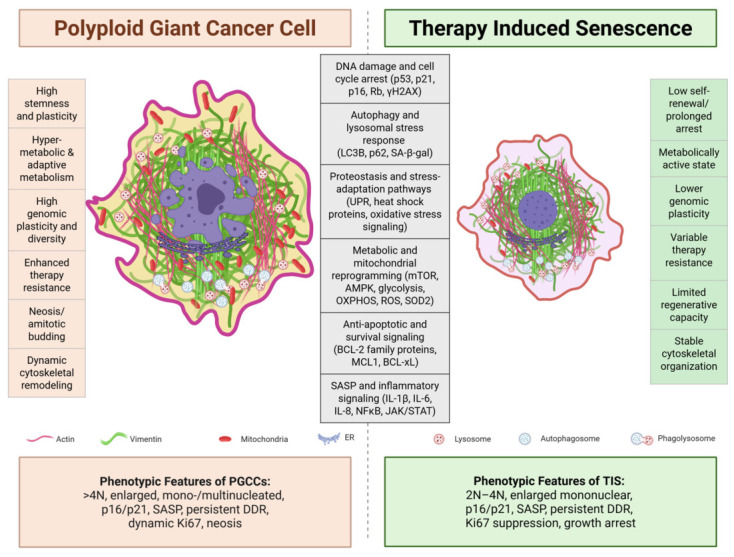
Shared and distinct biological features, phenotypic markers, and stress-associated pathways in polyploid giant cancer cells (PGCCs) and senescent cells. PGCCs are characterized by high stemness and cellular plasticity, hypermetabolism, extensive genetic diversity, therapy resistance, neosis-associated progeny generation, and dynamic cytoskeletal remodeling. In contrast, senescent cells typically exhibit low self-renewal capacity, prolonged but relatively stable growth arrest, lower genomic plasticity, variable therapy resistance, and more stable cytoskeletal organization. PGCCs are commonly associated with high-ploidy states (>4N), whereas therapy-induced senescent cells are more frequently associated with diploid to tetraploid states (2N–4N). Despite these distinctions, both PGCCs and senescent cells can exhibit persistent DNA damage signaling, p16/p21 pathway activation, SASP-associated inflammatory signaling, autophagy and lysosomal activation, altered metabolic adaptation, and anti-apoptotic stress-response pathways that promote survival under therapy-induced stress conditions. However, unlike canonically senescent cells, PGCCs retain the capacity for depolyploidization, neosis-associated progeny generation, and proliferative re-entry, which may be accompanied by reactivation of proliferative markers such as Ki67. Created in BioRender. Ghosh, D. (2026) https://BioRender.com/ocrrkf7 (9 May 2026).

Beyond their altered genomic state, PGCCs exhibit distinct structural and mechanical adaptations that support their survival and dissemination [12,51]. Their cytoskeleton is extensively reorganized, with reinforced actin stress fibers and intermediate filament networks that enhance force transmission, while increased nuclear plasticity enables migration despite their enlarged genomic content [12]. These cytoskeletal–nuclear adaptations support persistence in mechanically and metabolically challenging tumor environments. Shared and distinct structural, metabolic, and stress-adaptation features of PGCCs and therapy-induced senescent (TIS) cells are summarized in Figure 5. Although this section primarily focuses on PGCC biology, later sections directly compare PGCCs and TIS-associated pathways and therapeutic vulnerabilities.

Consistent with this, our work shows that PGCCs display increased stiffness, altered cytoskeletal organization, and slower but more persistent, directional migration [12]. Vimentin intermediate filaments (VIFs) form a diffuse, cytoplasm-spanning network in PGCCs, rather than the perinuclear bundling seen in non-PGCCs (Figure 4c), supporting structural integrity under elevated mechanical strain. In fact, the disruption of VIFs using acrylamide or VIM siRNA reduced cell volume, impaired motility, and abolished cell polarization, demonstrating that VIF organization was essential for maintaining PGCC structure and migratory persistence in MDA-MB-231 breast cancer cells [51].

These structural adaptations translate into a persistence-dominant mode of migration, enabling greater net displacement over time despite reduced speed [12,51]. Consistent with this, our studies showed that PGCCs become progressively enriched within scratch wounds (Figure 4c), exceeding levels predicted by their initial abundance in the population. This enhanced migratory persistence, combined with their ability to survive prolonged stress and later regenerate proliferative progeny, likely contributes to metastatic dissemination and tumor relapse. Accordingly, PGCCs function as long-lived reservoirs capable of surviving therapeutic stress and regenerating heterogeneous tumor populations [53].

Persistent stress also promotes autophagy in PGCCs, which is functionally linked to cytoskeletal remodeling and metabolic adaptation [45,51]. Autophagy represents a conserved stress-response mechanism that degrades and recycles damaged cellular components to maintain cellular homeostasis under conditions of prolonged stress. PGCCs use autophagy, including mitophagy and lipophagy, to recycle damaged components, limit oxidative stress, and sustain the metabolic demands of their enlarged size and migratory behavior [43,44,45,54]. By breaking down damaged cellular components, these pathways can generate metabolic precursors that support oxidative phosphorylation (OXPHOS) and ATP production during stress adaptation. This metabolic reprogramming is often accompanied by increased expression of antioxidant enzymes such as SOD2, which reduce mitochondrial reactive oxygen species (ROS) and further promote PGCC survival under hypoxic and therapy-induced stress conditions [55,56]. These metabolic alterations are highlighted in Figure 5. These processes are supported by a dispersed VIF network that may help organize autophagic machinery. Notably, disruption of either vimentin organization or autophagy reduces migratory persistence, highlighting a vimentin–autophagy axis that is critical for PGCC survival and dissemination in MDA-MB-231 breast cancer and HEY ovarian cancer cells [45]. These cytoskeletal and metabolic adaptations likely support not only PGCC survival during therapy-induced stress, but also the transition from prolonged stress-associated arrest toward regenerative escape and tumor repopulation.

## 4. PGCCs in Therapy Resistance and Tumor Recurrence

Chemotherapy, radiation, and other microenvironmental stressors induce extensive DNA damage, mitotic stress, oxidative stress, and metabolic dysfunction, which can disrupt spindle assembly and cytokinesis, leading to mitotic failure and the formation of polyploid giant cancer cells (PGCCs) [57,58]. Under these conditions, PGCCs can arise through mitotic slippage, endoreplication, or cell fusion, which enables cells to bypass cell death despite significant DNA damage and genomic instability (Figure 3) [9,44,46]. Their ability to tolerate additional microenvironmental stressors, including hypoxia, inflammation, and nutrient limitation, further enhances their survival and persistence in therapy-resistant tumors [54,58,59]. These stress-adapted properties may allow PGCCs to persist in prolonged dormant-like states before re-entering the cell cycle and contributing to tumor recurrence.

While previous studies on PGCCs have largely focused on their roles in chemoresistance and tumorigenicity, increasing evidence suggests that interactions between cancer cells and the surrounding microenvironment are also critical regulators of PGCC persistence, survival, and regenerative capacity. Cancer–stromal interactions promote drug resistance and metastasis through both paracrine signaling and direct cell–cell contact [60]. For example, marrow stromal cells (MSCs) enhance cancer cell proliferation and metastasis by inducing EMT via secreted factors such as TGF-β, IL-6, and IL-8 [18,61], while direct interactions can further confer drug resistance through integrin-mediated signaling [62].

Consistent with this paradigm, we observed that PGCCs are frequently located in close proximity to non-PGCCs under both naïve and paclitaxel-treated conditions (Figure 4d). Notably, non-PGCCs adjacent to PGCCs exhibited increased survival following PTX treatment, suggesting a protective effect [12,52]. These observations indicate that PGCCs may help establish localized chemoresistant niches, potentially through enhanced cell–cell interactions and signaling mechanisms analogous to those of stromal cells [43]. In addition, mechanically altered niches characterized by ECM stiffening, altered integrin signaling, and cytoskeletal tension may further support PGCC survival and stress adaptation.

PGCCs also display stem-like characteristics, including the ability to generate heterogeneous progeny and repopulate tumors following treatment [43]. This capacity for cellular plasticity links polyploidy to tumor regeneration, therapy resistance, and disease recurrence, and PGCCs are increasingly recognized as contributors to poor clinical outcomes [63].

Although numerous studies demonstrate strong associations between PGCCs and therapy-resistant phenotypes, including the ability of PGCCs to survive treatment and generate resistant progeny, definitive evidence establishing whether PGCCs are universally necessary or sufficient for therapy resistance across cancer types remains limited. Nevertheless, accumulating evidence supports the hypothesis that PGCCs may function as a major adaptive survival strategy capable of driving long-term resistance and recurrence in at least some contexts. At the same time, other resistant cellular states and parallel stress-adaptation programs likely also contribute to therapeutic failure and disease progression.

## 5. Evidence That Senescent Niches Promote PGCC Formation

Senescent and polyploid cells represent distinct outcomes of cellular stress and DNA damage, both capable of inducing cell-cycle arrest, often in a p53-dependent manner [64]. While senescence is traditionally defined as a stable and irreversible arrest, polyploid cells retain the capacity to re-enter the cell cycle through ploidy reduction (neosis), generating proliferative daughter cells that can repopulate tumors [46]. This reversible arrest enables PGCCs to survive toxic stress while sustaining the elevated metabolic and biosynthetic demands of their enlarged size and cellular architecture. Key features of PGCCs and senescent cells are illustrated in Figure 5.

Cancer therapies such as paclitaxel, doxorubicin, cisplatin, and radiation can enrich for both senescent cells and PGCCs in vitro and in vivo, suggesting overlapping adaptive programs that promote survival under cytotoxic stress [43,44]. PGCCs share several hallmarks of senescence, including enlarged morphology, growth arrest, oxidative stress responses, and expression of senescence markers such as p16, p21, and β-galactosidase [1,13,46]. Like senescent cells, PGCCs upregulate anti-apoptotic proteins, such as Bcl-2 and Mcl-1, promoting survival under extreme stress conditions, including hypoxia and high-dose chemotherapy [47]. They also develop a senescence associated secretory profile characterized by increased IL-6 and IL-8 production.

Aging tissues exhibit a progressive accumulation of polyploid cells across multiple organ systems, including liver, vascular, epithelial, and brain tissues, supporting the idea that polyploidy represents an evolutionarily conserved stress-adaptation program [37]. In many contexts, low-ploidy or tetraploid states appear to function as protective responses to genomic damage, oxidative stress, and tissue injury by limiting proliferation and buffering against stress-induced cell death [38]. For example, vascular smooth muscle cells in the aging aorta exhibit increased polyploidy associated with elevated Nox4, while hepatocytes undergo progressive polyploidization during aging and degeneration [40]. Similarly, luminal uroepithelial umbrella cells exhibit features consistent with “tetraploidy-induced senescence,” including SA-β-gal activity and persistent senescence-associated phenotypes, suggesting that stable polyploid states may function as long-term protective adaptations in certain tissues [65]. Experimentally induced tetraploid cells also frequently undergo p16INK4a-dependent growth arrest and acquire senescence-associated characteristics [38]. Together, these observations support the concept that stable tetraploid states may initially function as dormancy- or senescence-like barriers that preserve tissue integrity and limit proliferation of genomically unstable cells.

However, chronic aging-associated stress, persistent inflammation, or acute therapeutic stressors such as chemotherapy, radiation, hypoxia, or smoking-related damage may promote transition from stable tetraploid arrest toward more plastic and regenerative polyploid states associated with PGCC formation [10]. This transition from stable tetraploid arrest toward regenerative polyploid states may provide a conceptual link between therapy-induced senescence (TIS), dormancy-associated programs, and PGCC formation. Both TIS and PGCCs arise following genotoxic stress and share several stress-associated features, including persistent DNA damage signaling, p21 induction, SASP-associated cytokine secretion, SA-β-gal activity, and prolonged cell cycle arrest [42]. However, these states diverge in their long-term regenerative capacity. TIS is generally associated with stable proliferative arrest, sustained suppression of Ki67, and relatively organized p53/p21- or p16/Rb-associated signaling. In contrast, PGCCs exhibit greater cellular plasticity, progressive increases in ploidy, abnormal γH2AX signaling, altered metabolic and mechanobiological adaptation, and the capacity to undergo depolyploidization and neosis-like division. Importantly, Ki67 can become reactivated during progeny formation, suggesting that PGCCs may transition from dormancy-like arrest back into proliferative tumor regeneration [42,66].

These distinctions are highly relevant to tumor recurrence, metastasis, and aging-associated disease progression. PGCCs retain many features associated with dormant cancer cells, including stress tolerance, prolonged cell cycle arrest, and activation of p38/MAPK-associated dormancy pathways, while simultaneously maintaining enhanced migratory and invasive potential [66]. Aging-associated tissue microenvironments may further facilitate escape from dormancy and promote PGCC-driven recurrence. In melanoma, for example, the aged lung microenvironment promotes metastatic outgrowth, whereas younger lungs favor dormancy-associated signaling programs. Factors within aged fibroblast secretomes, including PROS1 and sFRP1, suppress dormancy-associated pathways and facilitate tumor reactivation [67]. Together, these findings support a model in which polyploidy initially functions as a stress-induced survival and dormancy-associated adaptation, but progressive genomic instability, depolyploidization, and microenvironmental reprogramming enable escape from arrest, tumor regeneration, and metastatic recurrence.

These similarities suggest that PGCCs represent a senescence-linked state; however, unlike canonical senescence, this state is not terminal and can give rise to proliferative progeny (illustrated in Figure 5). This reversibility suggests that PGCC fate is governed by dysregulated cell-cycle and mitotic control mechanisms that determine whether cells remain arrested or regain proliferative capacity [43,68]. One key regulator of this balance is Survivin (BIRC5), a mitotic protein that plays a central role in coordinating chromosome segregation and cytokinesis [69]. Its loss disrupts cytokinesis, producing polyploid cells with mitotic defects and DNA damage that activate a p53–p21–dependent senescence response [69,70]. Because Survivin levels must be tightly regulated, partial loss promotes genomic instability, whereas re-expression can enable cell-cycle re-entry and escape from senescence, contributing to tumor recurrence [71].

**Figure 6:**
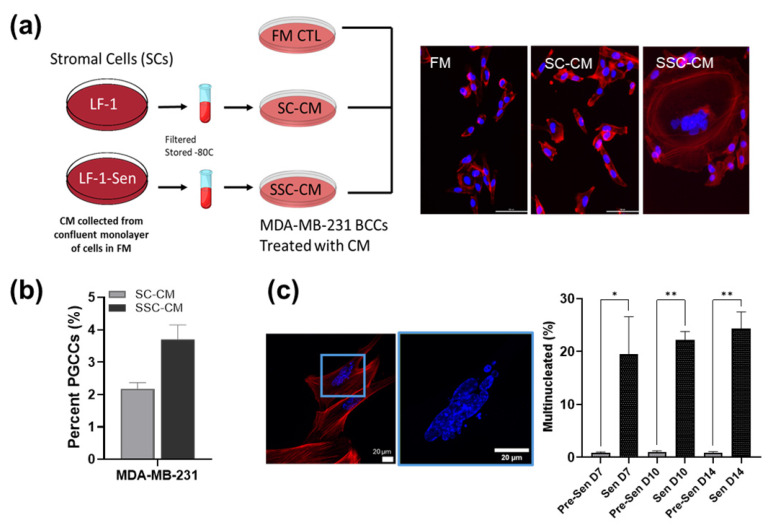
Senescent niches and therapeutic stress enhance PGCC formation. (**a**) MDA-MB-231 breast cancer cells were cultured in serum-free fresh media (FM) or serum-free conditioned media from pre-senescent (SC-CM) or senescent (SSC-CM) LF1 fibroblasts for 24 h, followed by staining for F-actin (phalloidin, red) and nuclei (Hoechst). (**b**) PGCCs were quantified based on their increased nuclear area. While SC-CM had little effect at 24 h, SSC-CM significantly increased PGCC frequency. (**c**) Increased frequencies of multinucleated stromal cells were also observed under senescence-associated ECM remodeling conditions in fibroblast-derived matrix models, suggesting that senescent niches broadly promote polyploidization across both cancer and stromal compartments. (* *p*  <  0.05, ** *p*  <  0.01). Parts of this figure were reproduced and modified from Xuan et al., Scientific Reports (2018) [12] and Howes et al., APL Bioengineering (2024) [23], both were distributed under Creative Commons Attribution (CC BY) licenses.

Our studies provide direct evidence linking senescent stromal environments to PGCC formation (Figure 6). Conditioned media from irradiated senescent fibroblasts (SSC-CM) induced a marked increase in enlarged, multinucleated MDA-MB-231 breast cancer cells (Figure 6a,b) [12,18]. These effects were not observed with conditioned media from proliferative fibroblasts (SC-CM), implicating SASP signaling in promoting PGCC emergence.

In complementary experiments, senescence-associated extracellular matrix remodeling increased the proportion of multinucleated cells to ~25% in LF1 lung fibroblasts, compared with <1% in pre-senescent conditions (Figure 6c) [23]. Together, these findings indicate that both paracrine signaling and matrix remodeling within senescent niches promote the formation of polyploid and multinucleated cells, likely through mitotic failure. Notably, this response is not restricted to cancer cells, as senescent stromal cells themselves can acquire polyploid phenotypes, which may further contribute to cancer progression and therapy resistance.

Mechanistically, SASP cytokines may promote aberrant cell-cycle progression, increasing the likelihood of mitotic slippage and failed cytokinesis [12,18,61]. At the same time, the dense and disordered extracellular matrix generated by senescent stromal cells may elevate cytoskeletal tension and disrupt mitotic spindle organization, promoting mitotic catastrophe and polyploidization [18,23]. Thus, senescent niches likely enrich for PGCC populations through a combination of biochemical and biomechanical stress signals that reshape the tumor microenvironment.

## 6. Therapeutic Vulnerabilities Shared Between Senescent Cells and PGCCs

**Figure 7:**
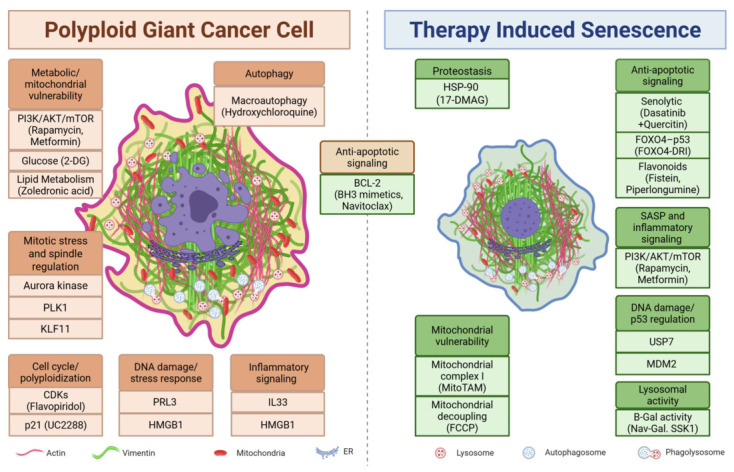
Shared and distinct therapeutic strategies targeting senescence-associated pathways and PGCC survival mechanisms. Current therapeutic approaches targeting PGCCs focus on metabolic and stress-adaptation pathways, including PI3K/AKT/mTOR signaling, glucose and lipid metabolism, mitotic stress pathways, cell-cycle regulation, DNA damage responses (DDR), inflammatory signaling, and autophagy. Because conventional chemotherapy and spindle-targeting agents can themselves promote PGCC formation through mitotic stress and mitotic slippage, combination approaches involving Aurora kinase or PLK1 inhibitors together with chemotherapy have emerged as promising strategies for suppressing therapy-induced polyploidization and regenerative escape. Additional approaches target CDKs, p21-associated signaling, autophagy, and inflammatory mediators linked to PGCC survival and neosis. In contrast, therapeutic strategies targeting therapy-induced senescence (TIS) primarily focus on senolytic or senomorphic approaches involving proteostasis pathways (HSP90 inhibitors), anti-apoptotic signaling (Navitoclax, Dasatinib + Quercetin (DQ)), SASP/inflammatory signaling, lysosomal activity (Nav-Gal, SSK1), and mitochondrial vulnerabilities (MitoTam, FCCP). Shared therapeutic vulnerabilities between PGCCs and senescent cells include dependence on anti-apoptotic BCL2 family proteins, metabolic adaptation, autophagy, and stress-associated survival signaling pathways. Created in BioRender. Ghosh, D. (2026) https://BioRender.com/ocrrkf7 (9 May 2026).

Therapeutics targeting senescence can be broadly categorized into senolytics, which selectively eliminate senescent cells, and senomorphics or senomodulators, which suppress senescence-associated phenotypes such as the pro-inflammatory senescence-associated secretory phenotype (SASP), often without inducing cell death [72]. Many senolytic strategies target enhanced pro-survival signaling pathways that allow senescent cells to resist apoptosis, including BCL-2 family proteins, p53 regulatory networks, and mitochondrial metabolic adaptations, as exemplified by agents such as combination therapy with Dasatinib and Quercetin (DQ), Navitoclax, and FOXO4-DRI [73]. Other approaches exploit hallmark features of senescent cells, including altered epigenetic regulation, elevated lysosomal β-galactosidase activity, chronic DNA damage signaling, and dysregulated mitochondrial function, including lysosome-directed therapeutics such as Nav-Gal and mitochondrial-targeted compounds such as MitoTAM [72]. In parallel, senomorphic agents such as rapamycin, metformin, and JAK/STAT or p38/MAPK inhibitors suppress inflammatory cytokine production, often through NF-κB-associated pathways, and reduce the metabolic dysfunction associated with the SASP, thereby limiting tissue damage and chronic inflammation linked to aging and senescent cell accumulation [73]. Emerging next-generation strategies, including Proteolysis Targeting Chimeras (PROTACs), offer increased specificity by selectively degrading senescence-associated proteins such as BCL2 or MDM2, while other therapeutics exploit metabolic and mitochondrial vulnerabilities that are enriched in senescent cells [72]. Because PGCCs share several stress-adaptation and survival pathways with senescent cells, increasing attention has focused on whether senolytic and senomorphic strategies may also be adapted to target therapy-resistant PGCC populations. Shared and distinct therapeutic strategies targeting senescence-associated pathways and PGCC survival mechanisms are summarized in Figure 7.

PGCCs possess unique biological vulnerabilities that have emerged as promising therapeutic targets in treatment-resistant and recurrent cancers. Current strategies focus on preventing the initial formation of PGCCs, disrupting survival during polyploid states, or blocking depolyploidization and progeny generation [47,54,74]. Importantly, many conventional chemotherapies and spindle-targeting agents, including paclitaxel and vincristine, can themselves promote PGCC formation through mitotic stress and mitotic slippage, necessitating combination approaches that suppress therapy-induced polyploidization while enhancing cancer cell killing. Accordingly, therapeutic strategies targeting mitotic regulators such as Aurora kinase, PLK1, HDAC, or CDK pathways, including agents such as flavopiridol, have shown promise in enhancing mitotic catastrophe, suppressing endoreplication, and preventing survival of polyploid cells generated during chemotherapy [47,74]. PGCCs also exhibit elevated metabolic demands, increased glycolysis, altered lipid metabolism, and heightened dependence on autophagy, making metabolic and stress-response pathways attractive therapeutic targets. Consequently, mTOR inhibitors (rapamycin, PP242/Torkinib), AMPK activators (resveratrol, salicylate), glycolytic inhibitors (2-deoxy-D-glucose), and autophagy-modulating drugs such as hydroxychloroquine have demonstrated potential for reducing PGCC survival, particularly when combined with cytotoxic therapies [54]. Additional vulnerabilities include dysregulated DNA damage responses, elevated anti-apoptotic signaling through BCL2 family proteins, ceramide metabolism, and senescence-associated pathways such as p21 signaling, which may be targeted using BH3 mimetics such as Navitoclax, PRL3-zumab, or p21 inhibitors such as UC2288 [74,75]. Emerging evidence also suggests that modulation of inflammatory or microenvironmental signals, including IL-33 and HMGB1, may suppress stress-induced polyploidization and neosis [74]. Together, these studies support the concept that PGCCs represent a distinct and targetable stress-adapted cellular state that contributes to chemoresistance, tumor dormancy, and metastatic recurrence.

Despite important biological differences, PGCCs and senescent cells share several overlapping stress-response pathways and therapeutic vulnerabilities, suggesting that senolytic and senomorphic therapies targeting these pathways may be adaptable for eliminating or reducing PGCC populations. Both cell states exhibit persistent DNA damage signaling, apoptosis resistance, dysregulated stress responses, altered epigenetic regulation, increased lysosomal activity, and secretion of pro-inflammatory cytokines and growth factors associated with the SASP [42]. PGCCs also exhibit several senescence-associated characteristics, including persistent growth arrest, p21 induction, SA-β-gal activity, and stress-associated inflammatory signaling [13]. Consequently, both senescent cells and PGCCs exhibit dependence on anti-apoptotic signaling pathways involving BCL2 family proteins, heightened autophagy, metabolic adaptation, and stress-response pathways, creating shared vulnerabilities that may be targeted using BH3 mimetics, autophagy inhibitors, metabolic modulators, and senolytic approaches such as Navitoclax [75].

Despite these similarities, important distinctions exist between senescent cells and PGCCs that influence their therapeutic targeting. Unlike canonically senescent cells, PGCCs retain regenerative potential through depolyploidization and neosis-like progeny formation, thereby contributing to tumor progression and recurrence. Their increased chromosome content and altered mitotic machinery create additional vulnerabilities involving Aurora kinase, PLK1, spindle assembly pathways, and metabolic stress tolerance that are less relevant in conventional senescence [47,76]. Furthermore, some signaling pathways associated with senescence may have paradoxical roles in PGCC biology. For example, factors such as p21, ROS, and Cdk1 can contribute both to senescence-associated growth arrest and to long-term PGCC survival following chemotherapy-induced stress [77]. Similarly, inflammatory signaling may produce distinct outcomes in PGCCs. IL-1β is a major component of the SASP and not only accumulates during senescence but can also actively reinforce senescence-associated signaling programs in neighboring cells. However, IL-1β inhibition combined with docetaxel treatment has been reported to paradoxically enhance PGCC formation, suggesting that inflammatory and stress-response pathways may function differently in polyploid stress-adapted states compared with canonical senescence [78]. Emerging studies also suggest that manipulation of nuclear envelope dynamics and lamin signaling, including induction of progerin-associated senescence programs, may selectively impair PGCC survival or regenerative capacity [78]. Together, these findings suggest that PGCCs represent a stress-adapted cellular phenotype that shares substantial overlap with senescence while retaining regenerative and tumor-promoting properties not typically associated with irreversible senescence.

Together, these findings support the concept that PGCCs represent a highly plastic stress-adapted cellular phenotype with overlapping but distinct biological and therapeutic vulnerabilities compared with canonical senescence. Increasing evidence suggests that effective therapeutic strategies may require combinatorial approaches that simultaneously target senescence-associated survival programs, mechanotransduction pathways, metabolic adaptation, and regenerative escape mechanisms that enable PGCC persistence and tumor repopulation following therapy-induced stress.

## 7. Conclusions and Future Directions

**Figure 8:**
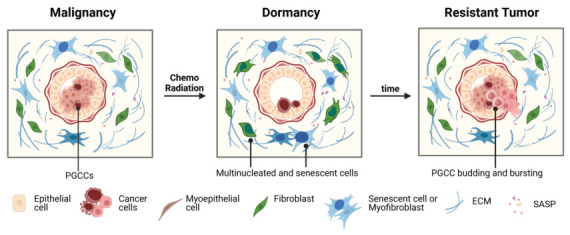
Senescence-driven ECM remodeling reprograms the tumor microenvironment to promote PGCC formation and tumor recurrence. Aging and cancer therapies induce senescence in stromal cells, triggering SASP signaling and extensive extracellular matrix (ECM) remodeling. These biochemical and mechanical changes reprogram the tumor microenvironment to promote cancer cell polyploidization and the emergence of polyploid giant cancer cells (PGCCs). While most tumor cells are eliminated by therapy, PGCCs evade treatment and persist in a dormant state within the tissue. These therapy-resistant cells later re-enter the cell cycle and generate aggressive progeny that seed tumor recurrence and metastasis. Created in BioRender. Dawson, M. (2026) https://BioRender.com/65w5a4x (27 March 2026).

Together, these studies support an emerging model in which senescence-associated remodeling of the tumor microenvironment likely contributes to the formation, persistence, and regenerative potential of PGCCs through coordinated biochemical, inflammatory, metabolic, and mechanobiological signaling pathways. Senescent stromal cells reshape the tumor niche through SASP signaling, extracellular vesicle secretion, extracellular matrix remodeling, and altered cytoskeletal tension, creating conditions that enhance cancer cell plasticity, stress adaptation, and polyploidization. This conceptual framework is summarized in Figure 8.

Cellular senescence and polyploid giant cancer cells (PGCCs) are increasingly recognized as interconnected stress-adaptation programs that emerge in response to aging, genotoxic injury, metabolic stress, and cancer therapy. While senescence has historically been viewed as a tumor-suppressive process and polyploidy as a consequence of mitotic failure, growing evidence suggests that these states share overlapping biological features, mechanotransduction pathways, metabolic adaptations, and inflammatory signaling programs that can promote tumor persistence under extreme stress conditions. Importantly, PGCCs are not simply enlarged or terminally damaged cancer cells, but rather highly plastic stress-adapted cellular states capable of surviving therapy-induced stress, persisting in dormant-like conditions, and later regenerating proliferative progeny through depolyploidization and neosis. These properties position PGCCs at the intersection of therapy-induced senescence, dormancy, stemness-associated reprogramming, and tumor recurrence.

At the same time, important biological distinctions remain between canonical senescence and PGCC biology, particularly regarding reversibility, regenerative potential, genomic plasticity, and mitotic re-entry (Figure 5). Because PGCCs share morphological and molecular features with senescent cells, they may also be difficult to distinguish within the tumor microenvironment, potentially enabling them to evade detection while retaining malignant potential [13,46,79]. Defining these distinctions more clearly and identifying reliable biomarkers for PGCC detection, especially in clinical samples, will be essential for understanding how stress-adapted tumor cell populations contribute to metastatic progression and therapeutic resistance.

Despite increasing recognition of PGCCs in aggressive and recurrent cancers, many important questions remain unresolved. Mechanistic studies directly linking senescence-associated extracellular matrix remodeling, mechanotransduction signaling, inflammatory SASP factors, and PGCC formation remain limited. Future studies involving direct manipulation of matrix stiffness, mechanotransduction pathways, and specific SASP-associated inflammatory factors will be necessary to establish causal relationships between senescence-associated niche remodeling and PGCC formation, persistence, and regenerative escape. Future studies integrating lineage tracing, spatial transcriptomics, single-cell multiomics, live-cell imaging, and biomechanical profiling approaches will likely be critical for defining the temporal emergence, reversibility, and functional significance of PGCC states during therapy response and tumor evolution. Collectively, these approaches may help clarify how PGCCs contribute to cancer progression and determine the extent to which PGCC-associated stress-adaptation programs are necessary or sufficient for long-term therapy resistance and tumor recurrence across different cancer types.

The growing recognition that PGCCs share therapeutic vulnerabilities with senescent cells also creates new translational opportunities. Therapeutic strategies targeting anti-apoptotic signaling, autophagy, mechanotransduction pathways, metabolic adaptation, depolyploidization, and stress-associated inflammatory signaling may provide new approaches for preventing tumor recurrence and eliminating therapy-resistant residual disease. Together, these findings highlight PGCCs as an important therapeutic and conceptual frontier in cancer biology and suggest that disrupting senescence-associated niches or eliminating PGCC survival pathways may ultimately help eradicate dormant cancer cell reservoirs and improve long-term treatment outcomes.

## Figures and Tables

**Table 1:** Confirmation of senescence in human MSCs and lung fibroblasts.

	Human MSCs	LF1 Fibroblasts
β-galactosidase positive ^1^	38.1	18.5
BrDU positive ^1^	0.1	0.1
Cell Area ^1^	4.2	6.4
Nuclear Area ^1^	1.6	1.5
Cell Velocity ^1^	0.7	0.8
P16 ^2^	1.59	3.32
P21 ^2^	2.09	2.44
APO1 ^2^	1.79	3.08

^1^ Fold-change for senescent vs. pre-senescent cells, determined from published data. ^2^ Log2 fold-change for senescent vs. pre-senescent cells normalized to β-actin, determined from published data. Both cell types were irradiated (15 Gy) and cultured for 10 days to induce senescence. This table was generated from data included in Ghosh et al., Journal of Cell Science (2020) [18], and Howes et al., APL Bioengineering (2024) [23].

## Data Availability

No new data were created for this manuscript.

## Abbreviations

The following abbreviations are used in this manuscript:

AMPK	AMP-activated protein kinase
BCL-xL	B-cell lymphoma-extra large
CDK	Cyclin-dependent kinase
DDR	DNA damage response
DQ	Dasatinib + quercetin
ECM	Extracellular matrix
EV	Extracellular vesicle
FAK	Focal adhesion kinase
HMGB1	High mobility group box 1
HSP90	Heat shock protein 90
IL	Interleukin
MCL1	Myeloid cell leukemia 1
MSC	Marrow stromal cell
mTOR	Mechanistic target of rapamycin
NFκB	Nuclear factor kappa B
OXPHOS	Oxidative phosphorylation
PGCC	Polyploid giant cancer cell
PI3K	Phosphoinositide 3-kinase
PLK1	Polo-like kinase 1
PROTAC	Proteolysis targeting chimera
PTX	Paclitaxel
ROCK	Rho-associated protein kinase
ROS	Reactive oxygen species
SA-β-gal	Senescence-associated β-galactosidase
SASP	Senescence-associated secretory phenotype
SHG	Second harmonic generation
SOD2	Superoxide dismutase 2
TGFβ	Transforming growth factor beta
TIS	Therapy-induced senescence
TNBC	Triple-negative breast cancer
UPR	Unfolded protein response
VIF	Vimentin intermediate filament
VIM	Vimentin
γH2AX	Phosphorylated H2AX histone marker
